# Low Concentrations of Caffeine and Its Analogs Extend the Lifespan of *Caenorhabditis elegans* by Modulating IGF-1-Like Pathway

**DOI:** 10.3389/fnagi.2018.00211

**Published:** 2018-07-16

**Authors:** Xiaocui Du, Yun Guan, Qin Huang, Ming Lv, Xiaofang He, Liang Yan, Shuhei Hayashi, Chongye Fang, Xuanjun Wang, Jun Sheng

**Affiliations:** ^1^Key Laboratory of Pu-erh Tea Science, Ministry of Education, Yunnan Agricultural University, Kunming, China; ^2^Tea Research Center of Yunnan, Yunnan Academy of Agricultural Sciences, Kunming, China; ^3^College of Food Science and Technology, Yunnan Agricultural University, Kunming, China; ^4^Pu’er Institute of Pu-erh Tea, Pu’er, China; ^5^Department of Microbiology, Hyogo College of Medicine, Nishinomiya, Japan; ^6^China-Japan Joint Center for Bioresource Research and Development, Yunnan Agricultural University, Kunming, China; ^7^College of Veterinary Medicine, Yunnan Agricultural University, Kunming, China; ^8^State Key Laboratory for Conservation and Utilization of Bio-Resources in Yunnan, Kunming, China; ^9^College of Science, Yunnan Agricultural University, Kunming, China

**Keywords:** caffeine, *C. elegan*s, lifespan, IGF-1 pathway, *daf-2*

## Abstract

Caffeine has been reported to delay aging and protect aging-associated disorders in *Caenorhabditis elegans*. However, the effects of low concentration of caffeine and its analogs on lifespan are currently missing. Herein, we report that at much lower concentrations (as low as 10 μg/ml), caffeine extended the lifespan of *C. elegans* without affecting food intake and reproduction. The effect of caffeine was dependent on IGF-1-like pathway, although the insulin receptor homolog, *daf-2* allele, *e1371*, was dispensable. Four caffeine analogs, 1-methylxanthine, 7-methylxanthine, 1,3-dimethylxanthine, and 1,7-dimethylxanthine, also extended lifespan, whereas 3-methylxanthine and 3,7-dimethylxanthine did not exhibit lifespan-extending activity.

## Introduction

Caffeine (1,3,7-trimethylxanthine) has been reported to act through *daf-16* and *cbp-1* to increase the lifespan of the nematode model organism *Caenorhabditis elegans* (Lublin et al., 2011). Subsequent studies found that caffeine induced longevity by mimicking dietary restriction and modulating the insulin/IGF-1-like signaling (IIS) pathway (Bridi et al., 2015). Moreover, as the most important molecular target of caffeine *in vivo*, the adenosine receptor might be involved in the lifespan-prolonging impact of caffeine (Chen et al., 1999).

Habitual human consumption of caffeine-containing foods and beverages is estimated to be in the range of 70–350 mg/person/day or 5–8 mg/kg/day (equivalent to three cups of coffee). In human, higher doses (400–500 mg/day) of caffeine may lead to side effects, including increased anxiety, increased blood pressure, headache, and confusion (Chen et al., 2010). The concentrations used in previous studies which reported the life-span extending effect of caffeine ranged from 1 to 100 mmol/L (18 mmol/L = 1 g/L), which is relatively higher than physiological doses (Lublin et al., 2011; Sutphin et al., 2012; Bridi et al., 2015). Thus, studies examining the effects of low caffeine concentrations on life span are currently missing.

Caffeine, which is widely consumed in beverages, possesses stimulant properties due to the blockage of adenosine receptors (Chen et al., 1999; Fredholm et al., 1999; Temple, 2009). Other methylxanthines interact with adenosine receptors as well, and their affinities vary according to different adenosine receptor subtypes and the chemical structures of the methylxanthines. Because the adenosine receptor might be involved in the lifespan-extending effect induced by caffeine (Chen et al., 1999), it is also possible that these caffeine analogs extend the lifespan of *C. elegans*.

The IIS pathway is an evolutionarily conserved pathway that regulates metabolism, development, stress resistance, and lifespan (El-Tanani and Green, 1996; Duanmu et al., 1999; Ackerman and Gems, 2012; Matilainen et al., 2013; Heimbucher and Hunter, 2015). In *C. elegans*, the insulin-like receptor, *daf-2*, signals through a phosphoinositide 3 (PI3)-kinase (*age-1*/*aap-1*) (Gottlieb and Ruvkun, 1994) signaling cascade that activates downstream serine/threonine kinases including *pdk-1, akt-1, akt-2*, and *sgk-1*. These kinases in turn function to negatively regulate the transcription factor, *daf-16* (FOXO) to modulate the expression of downstream genes. High doses of caffeine (≥5 mmol/L) delayed larval development and increased lifespan, and these impacts were reversed when *daf-2* or *daf-16* was knocked-down by RNAi, which suggested that the effect of caffeine was dependent on the IIS pathway. In this study, we tested whether low concentrations of caffeine and its analogs could increase the lifespan of *C. elegans*.

## Materials and Methods

### Strains

All worm strains including *daf-2* (*e1370, e1371*), *age-1* (*hx546*), *daf-16* (*mu86*), *akt-1* (*ok525*), *akt-2* (*ok393*), and *daf-16* (*mu86*) mutants, as well as wild-type N2 (Bristol) and *daf-16*::GFP (*zls356*) strains, were obtained from the Caenorhabditis Genetics Center (University of Minnesota, Minneapolis, MN, United States).

### *C. elegans* Assays

All strains were grown at 20°C on standard Nematode Growth Medium (NGM) seeded with *Escherichia coli* strain *OP50* as the food source (Brenner, 1974).

### Caffeine Treatments

Sterilized caffeine stock solution was added into NGM and *OP50* to final concentrations as indicated. Plates were prepared the day before use. On the second day, 100 μl of bacterial solution was added to the control plate, and 100 μl of the bacterial solution containing 50-μg/ml caffeine was added to the treatment group.

### Lifespan Analysis

Lifespan analysis was performed at 20°C as previously described (Kenyon et al., 1993). The strains grew for at least two generations at 20°C without starvation prior to lifespan analysis. The total numbers of animals in the experiments are listed in **Tables 2, 3**. In all experiments, lifespan analysis was performed using the time of egg laying as *t* = 0 for synchronization.

### Body Length

During nematode development, body length can reflect the impact of drugs. In the nematode life cycle, drugs could play a role in promoting or delaying nematode spawning and development. In this study, we simultaneously cultured the treatment and control groups and examined their lengths. From the third day, 15 nematodes were randomly selected every 24 h from each group and anesthetized with 0.1% sodium azide delivered by a nematode gun. The nematode bodies were straightened and lengths determined under a microscope at 40× magnification and an eyepiece with a scale.

### Brood Size Assays

Each worm was allowed to lay eggs and then transferred to a new NGM plate every 24 h until the egg laying period was complete. Hatched worms were counted after 48 h of incubation at 20°C (Li et al., 2008). *C. elegans* that crawled off the plate, exploded or bagged were not examined (Hsu et al., 2003). Twelve *C. elegans* were used for each treatment condition. The experiment was repeated at least three times.

### Pharyngeal Pumping Rate Assay

In this study, the experimental and control group nematodes were simultaneously treated. Each group consisted of a certain number of synchronized nematodes. The time to swallow food was measured visually every 40 h. At each time, 15 nematodes were randomly selected from each group to determine the number of times they had swallowed (i.e., the number of times the pharyngeal muscles contracted) in 1 min at different time points.

### DAF-16::GFP Nuclear Localization

At 20°C, transgenic worms expressing DAF-16::green fluorescent protein (GFP) were transferred to a plate containing 0 or 50 μg/ml caffeine at the L4 developmental stage. The worms were fixed by treatment with 25 nmol/L NaN_3_. Still images were captured using a mounted digital camera and a fluorescence channel of an Olympus Ckx41 microscope. The captured images were used to quantify visible GFP lesions at 20°C (Sutphin et al., 2012).

### Quantitative RT-PCR Analysis

Fed worms were collected and RNA was prepared using TRIzol Reagent (Invitrogen, San Diego, CA, United States). After quantification, 1 μg of total RNA was used in a reverse-transcription reaction with SuperScript III (Invitrogen) to generate cDNA.

The PCR mixture consisted of 0.3 mM primers, cDNA, ROX, and 16 SYBR green mix (Invitrogen Platinum SYBR green qPCR Supermix UDG). The quantitative RT-PCR (qRT-PCR) was run with an ABI 7900. The level of each mRNA was analyzed using the ΔΔCt method and normalized to that of the corresponding *act-1*. Primer sequences were listed in **Table 1**.

**Table 1:** Primer sequences of genes for RT-PCR.

Primer name	Forward (5’–3’)	Reverse (5’–3’)
*act-1*	GCTGGACGTGATCTTACT GATTACC	GTAGCAGAGCTTCTCC TTGATGTC
*daf-1*	GGAGATCTCGGTCTCTCGTTATC	*TTCCGGTGCAAGGTATCTAAC*
*daf-2*	GAGAGAATGATGTGCCAACG	*CACCACGATTTGTTAGGCAATA*
*daf-3*	AGGGCACCAAGGTCAGGTA	*CCAAGGTGACTGTTGATGGTT*
*daf-4*	CAGGCCGTACTTCTTTGGAC	*TCACAAGAATGTTCTTC* *GACTTG*
*daf-16*	*CCAGGAAGGAATCCACGGCGT*	*TGGCTCCGCGGCGAGATTTT*
*daf-18*	*ACCAGGTCGATGGCTCGTGAC*	*TGGCGAATCCGCTCGACGATT*
*age-1*	*ACGAACCGCGTGCTCAATCG*	*TCTCTGCACGGAGCAGCCAG*
*akt-1*	*AGTCAGCTAAAGCATGGGAGA*	*TCTTTGATTTGACGTTCACTGG*
*cbp-1*	*ACGGAGGAAGAACGGGAAAC*	*TTCCCGCATCCTAAGCCAAG*
*mrp-5*	*GACTGTCAGGGGGCTACCTA*	*ATACAGGGGTCTCCACGACA*
*hpd-1*	*ATGGAGAAACCGACCACACC*	*ATGTGGCTGGCATTGGATGA*
*ins-7*	*TCCCTGCTTCTCAACAATATCC*	*TCGATTACTTCTTCATTGA* *TTATTTGA*
*pnk-la*	*CCGCTGCTACAGAGGATCAG*	*TCAGCAGCACATACTCGTGG*
*pnk-lb*	*CTGATTGCTGGATCAATGGCTAA*	*TGCCATACAAATATGCC* *ATCTGAAA*
*hsf-1*	*AGAGATGCGTGCGATGCGAGA*	*GGCGAGCATGTTGTTGACGCA*

### Western Blotting Analysis

Extracts from day 1 adult N2 *C. elegans* grown on control or caffeine containing NGM plates were harvested and washed twice with cold M9 buffer. The animals were then washed with homogeneous buffer (HB buffer, 20 mmol/L Hepes at pH 7.6, 100 mmol/L NaCl, 10 mmol/L KCl, 1.5 mmol/L MgCl_2_, 0.1 mmol/L ethylenediaminetetraacetic acid (EDTA), 0.5 mmol/L ethyleneglycol-bis (2-aminoethylether)-*N,N,N*’,*N*’-tetraacetic acid (EGTA), 44 mmol/L sucrose, and 0.5% Triton X-100). The pellet was re-suspended in 3× volumes of HB buffer with 1.5 mmol/L NaF, 2 mmol/L Na_2_VO_4_ and protease inhibitor cocktail (Roche, Basel, Switzerland). The worms were then lysed by two freeze-thaw cycles. The lysate was transferred to a pyrolysis instrument at a frequency of 30 times/s for 2 min. The lysate was collected and centrifuged at 14,000 × *g* for 20 min. The supernatant was collected and protein concentration determined by Bradford assay (Pierce, Thermo Fisher Scientific, Rockford, IL, United States). Briefly, tissue lysates were prepared from *C. elegans* using radioimmunoprecipitation assay (RIPA) buffer (Solarbio, Beijing, China) according to manufacturer protocols. Cell and tissue extracts were normalized to determine protein concentrations by the bicinchoninic acid method. Proteins were separated by sodium dodecyl sulfate–polyacrylamide gel electrophoresis and then transferred to polyvinylidene difluoride (PVDF) membranes (Millipore, Bedford, MA, United States). After gentle washing, blocking, and incubation with the following primary antibodies, AKT 1/2/3 (SC-8312, Santa Cruz), and p-AKT 1/2/3 (Thr308; sc-16646-R, Santa Cruz), PVDF membranes were incubated with the appropriate horseradish peroxidase-conjugated secondary antibody. Protein bands were detected using a Pro-light Horseradish Peroxidase Chemiluminescence kit (Tiangen Biotech, Beijing, China). Images were acquired with a FluorChem^TM^ E system (ProteinSimple, Santa Clara, CA, United States).

### Statistical Analysis

Statistical analysis was performed using GraphPad (version 5.0). The Kaplan–Meier method was applied to calculate survival fractions and log-rank (Mantel-Cox) test was used to compare survival curves. The log-rank (Mantel-Cox) test or one-way analysis of variance (ANOVA) followed by Bonferroni post-test was used to check for significant differences between means for other comparisons. *P*-values lower than 0.05 were considered significant.

## Results

### Low Concentration of Caffeine Extends Lifespan of *C. elegans* Bristol N2 Strain

**FIGURE 1:**
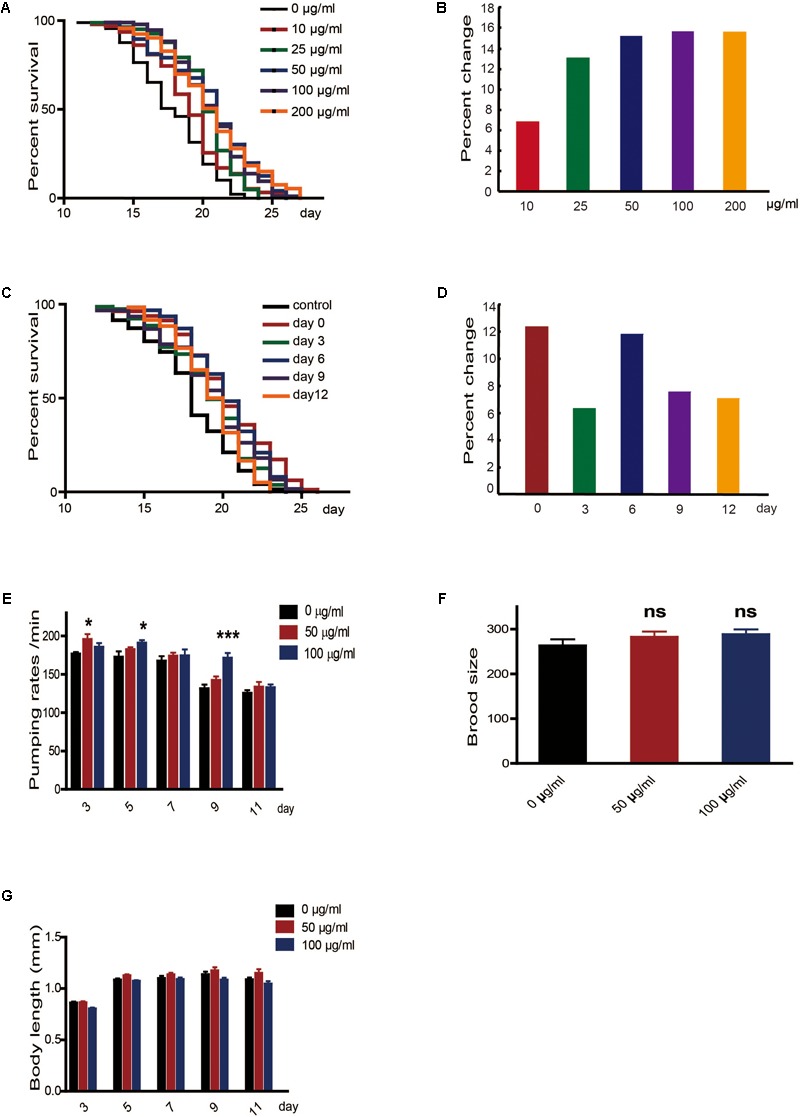
Low concentrations of caffeine increased *C. elegans*’ lifespan from the period of egg hatching. **(A,B)** Low concentration of caffeine increased *C. elegans’* lifespan in a dose-dependent manner. **(C,D)** Caffeine treatment at various time points extended lifespan. **(E)** 50 μg/ml and 100 μg/ml caffeine did not reduce pumping rate, and 15 worms were used for each group. **(F)** 50 μg/ml and 100 μg/ml caffeine did not affect brood size, and 15 worms were used for each group. **(G)** 50 μg/ml and 100 μg/ml caffeine did not affect body length (head-to-tail). The log-rank (Mantel-Cox) test was used to analyze the differences. Error bars, values are expressed as mean ± SEM; ^∗^*p* < 0.05; ^∗∗^*p* < 0.01; ^∗∗∗^*p* < 0.001.

To determine whether caffeine extended the lifespan of worms, we exposed N2 wild-type *C. elegans* to caffeine and measured their lifespans. Low concentrations of caffeine increased their lifespans in a dose-dependent manner (**Figures 1A,B**). The time course of the effect of caffeine was also tested. Caffeine at 100 μg/ml was fed to worms on days 0, 3, 6, 9, and 12, and the results indicated that caffeine was effective at all these time-points (**Figures 1C,D**), and its effect was time-dependent. To exclude the possibility that caffeine decreased food intake by the worms, the pumping rate was measured. The result showed that caffeine did not reduce food intake (**Figure 1E**), which suggested that caffeine did not prolong lifespan by calorie restriction. Inconsistent with previous reports (Bridi et al., 2015), low concentrations of caffeine did not affect reproduction and body length of the worms (**Figures 1F,G**, **Table 2**, and Supplementary Table S1).

**Table 2:** Effects of 50 μg/ml caffeine on lifespan (representative data).

Strain	Mean lifespan (days) (+caf/-caf)	*P*-value	Number of animals (+caf/-caf)
N2	20.09 ± 2.89/17.41 ± 2.91	*P* < 0.001	126/115
daf-2(e1371)	32.81 ± 5.23/29.38 ± 5.26	*P* < 0.001	92/85
daf-2(e1370)	40.44 ± 7.43/39.88 ± 7.27	*P* = 0.4155	124/116
age-1(hx546)	26.08 ± 3.95/25.98 ± 4.27	*P* = 0.5497	108/113
akt-1(ok525)	26.84 ± 4.06/26.78 ± 5.13	*P* = 0.5885	88/82
akt-2(ok393)	27.56 ± 3.88/27.69 ± 4.73	*P* = 0.2144	81/90
daf-16(mu86)	17.24 ± 2.05/17.26 ± 1.97	*P* = 0.8344	96/111

### Effect of Caffeine Was Dependent on IGF-1 Pathway

**FIGURE 2:**
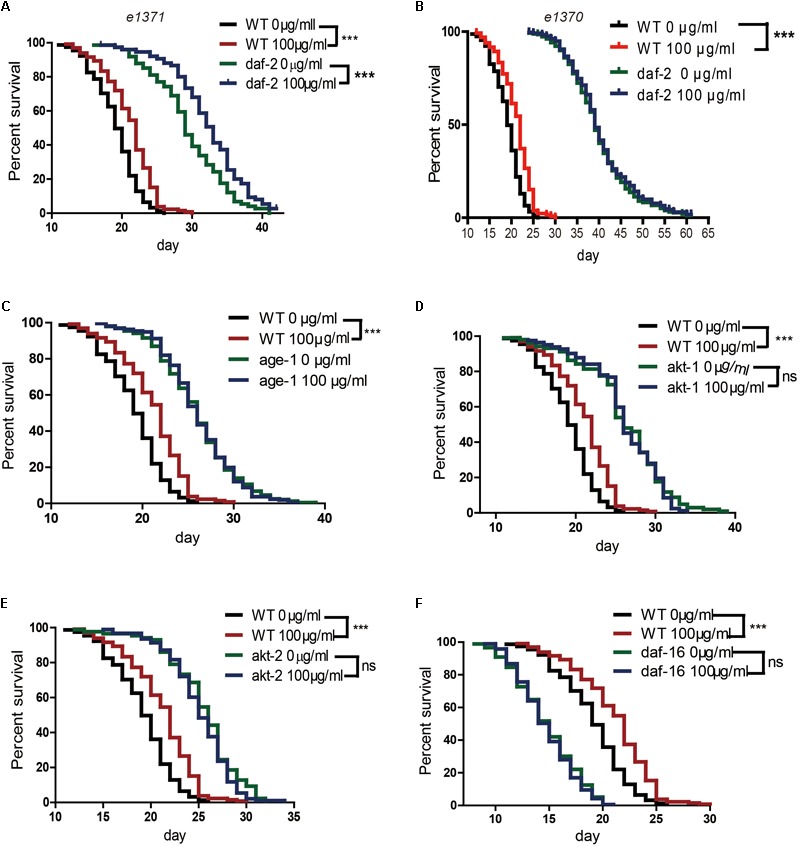
Effects of caffeine on lifespan from the time of egg hatching of N2 wild-type (WT) and IGF-1 pathway mutant strains. **(A)** N2 wild-type and *daf-2* (e1371) mutant strain; **(B)** N2 wild-type and *daf-2* (e1370) mutant strain; **(C)** N2 wild-type and *age-1* (hx546) mutant strain; **(D)** N2 wild-type and *akt1 (ok525)* mutant strain; **(E)** N2 wild-type and *akt-2 (ok393)* mutant strain; **(F)** N2 wild-type and *daf-16* (mu86) mutant strain. The log-rank (Mantel-Cox) test was used to analyze the differences. Error bars, values are expressed as mean ± SEM; ^∗^*p* < 0.05; ^∗∗^*p* < 0.01; ^∗∗∗^*p* < 0.001.

Several genetic pathways influence longevity and may mediate the lifespan-extending effects of caffeine. In worms, the insulin/IGF-1 signaling pathway is a major molecular pathway that influences lifespan (van Heemst, 2010). To investigate whether the insulin/IGF-1 pathway was involved in the lifespan extension effects of caffeine, *daf-2, age-1, akt-1, akt-*2, and *daf-16* loss-of-function mutants were exposed to caffeine and their lifespans evaluated. Similar to wild-type worms, caffeine extended the lifespan of *daf-2* (*e1371*) mutant worms (**Figure 2A**). However, in *age-1* (*hx546*), *akt-1* (*ok525*), *akt-2* (*ok393*), and *daf-16* (*mu86*) mutant strains, caffeine lost its effects on lifespan extension (**Figures 2C–F**). These results suggested that low concentrations of caffeine extended the lifespan of worms by modulating the IGF-1 pathway. The *daf-2* (*e1371*) mutation did not affect caffeine’s effect, suggesting that the role of *daf-2* (*e1371*) was not irreplaceable. However, caffeine did not further prolong life span in *daf-2* (*e1370*) mutant strain (**Figure 2B**), which was consistent with previous study (Bridi et al., 2015). Since *e1370* allele was a stronger mutation, these results suggested that caffeine’s effect was dependent on IGF-1 pathway.

### Acute Caffeine Treatment Induced DAF-16 Translocation to the Nucleus

**FIGURE 3:**
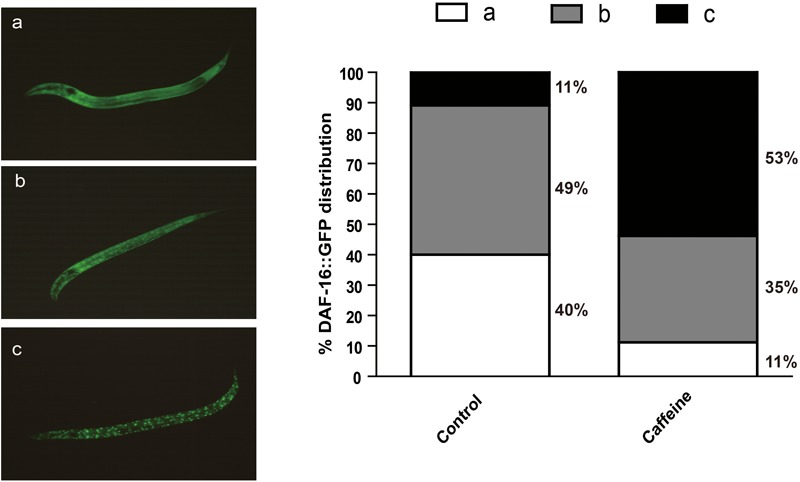
Acute caffeine (50 μg/ml) exposure translocates DAF-16 to the nucleus. The GFP localization status was characterized into three types defined as **(a–c)**, as shown in the figure. The numbers of each type were counted and percentages were calculated. *N* = 176 and 197, respectively.

The activity of daf-16 is regulated by subcellular localization. The reduction of IIS causes dephosphorylation and nuclear translocation of DAF-16 to activate transcription of target genes. To determine whether caffeine affects DAF-16 nuclear localization, we exposed GFP-labeled DAF-16 transgenic worm (*zls356*) to caffeine, and analyzed DAF-16 location. Animals exposed to caffeine showed higher DAF-16::GFP nuclear/cytoplasmic fluorescence ratios than vehicle-treated worms (**Figure 3**). These results confirmed that the effect of caffeine on lifespan was dependent on DAF-16, which is consistent with the results of mutant strains (**Figure 2**).

### Caffeine Modulated Lifespan-Related Gene Expression in *C. elegans*

**FIGURE 4:**
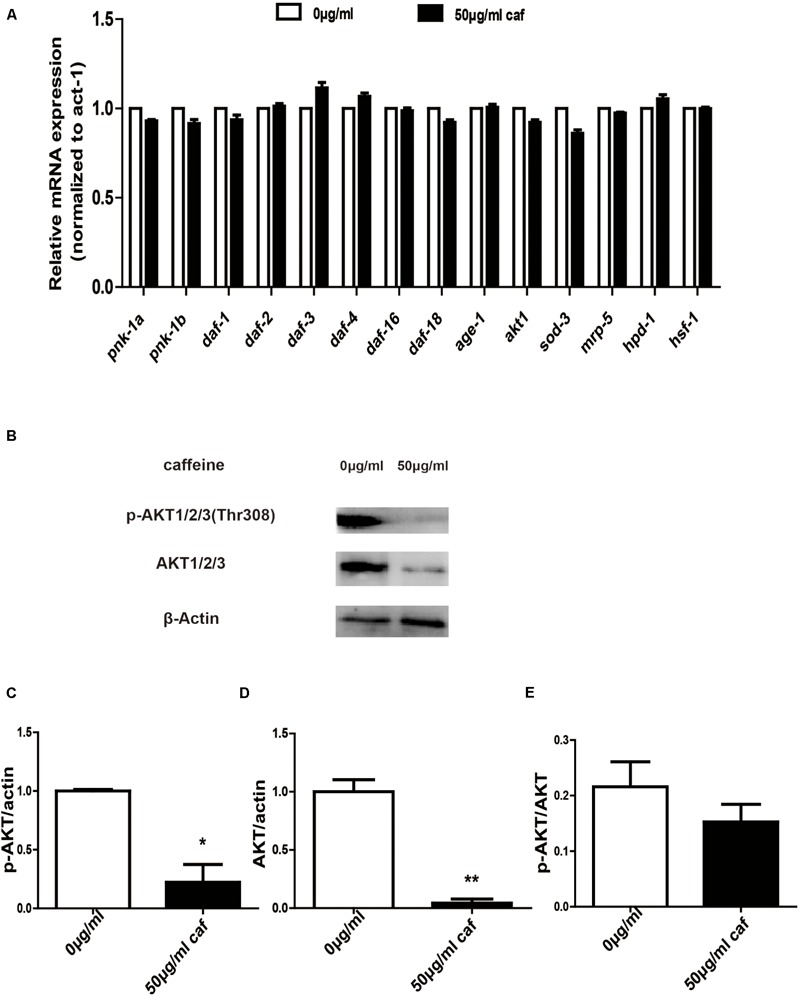
Caffeine modulated the AKT signaling pathway. **(A)** Caffeine increased longevity-related gene expression which acted downstream of AKT. **(B)** Caffeine prohibited expression and phosphorylation of AKT 1/2/3 (Thr308); **(C–E)** Quantification of expression and phosphorylation of AKT. One-way analysis of variance (ANOVA). Error bars, values are expressed as mean SEM; ^∗^*p* < 0.05; ^∗∗^*p* < 0.01.

In order to further verify that caffeine extended lifespans of nematodes, we extracted nematode RNA and protein and examined expression of lifespan-related genes and *akt* phosphorylation. We found that caffeine significantly promoted *daf-3, daf-4*, and *ins-7* mRNA expression in *C. elegans* (**Figure 4A**). Caffeine at 50 μg/mL inhibited AKT 1/2/3 expression and phosphorylation (**Figures 4B–E**), although the ratio of p-AKT/AKT was not significantly reduced. These results indicated that caffeine acted on AKT pathway and in turn promoted expression of downstream target genes.

### The Effect of Methyl Substitution at Different Sites of Xanthine on Longevity of *C. elegans*

**FIGURE 5:**
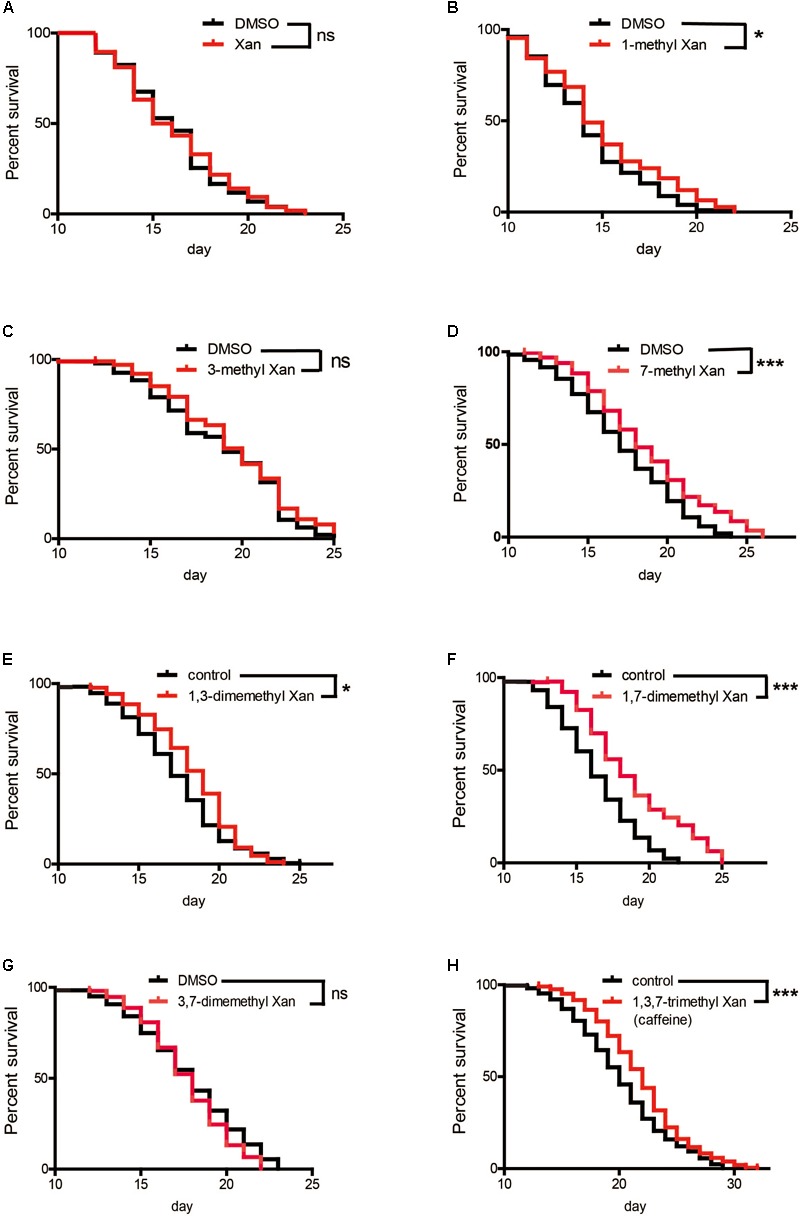
Effects of caffeine analogs on *C. elegans*’ lifespan from the period of egg hatching. **(A–H)** Four caffeine analogs, 1-methyl xanthine, 7-methyl xanthine, 1,3-dimethyl xanthine, and 1,7-dimethyl xanthine, also extended worm lifespan. Xanthine, 1-methyl xanthine, 3-methyl xanthine, 7-methyl xanthine, and 3,7-dimethyl xanthine were dissolved in DMSO to a final concentration of 0.1%, therefore 0.1% DMSO served as control. The log-rank (Mantel-Cox) test was used to analyze differences. Error bars, values are expressed as mean ± SEM; ^∗^*p* < 0.05; ^∗∗^*p* < 0.01; ^∗∗∗^*p* < 0.001.

Caffeine (1,3,7-trimethylxanthine) is a xanthine with three methyl groups. Xanthine failed to prolong worm’s lifespan (**Figure 5A**). To identify the role of these methyl groups in the chemical structure of caffeine, we tested the effect of several caffeine analogs on longevity of *C. elegans*. Four analogs of caffeine (1-methylxanthine, 7-methylxanthine, 1,3-dimethylxanthine, and 1,7-dimethylxanthine) also extended worm lifespan, whereas 3-methylxanthine and 3,7-dimethylxanthine failed to exhibit lifespan-extending activity. These results indicated that the 1-methyl group was more important for the effect of caffeine, because all analogs containing 1-methyl extended lifespan (**Figures 5B,E,F,H** and **Table 3**). The role of 7-methyl was unclear because 7-methylxanthine, but not 3,7-dimethylxanthine, was effective (**Figures 5D,G** and **Table 3**). However, 3-methyl appeared unnecessary for the effects of caffeine, because 3-methylxanthine and 3,7-dimethylxanthine did not extend lifespan (**Figures 5C,G** and **Table 3**).

**Table 3:** Effects of 50 μg/ml caffeine and its analogs on lifespan.

Compound	Number of experiments	Mean lifespan (days) (+caf/-caf)	Percentage change	*P*-value	Number of animals (+caf/-caf)
Xanthine	3	17.11 ± 2.34/16.97 ± 2.56	0.82	*P* = 0.6860	230/215
1-methyl Xanthine	3	16.35 ± 2.55/17.22 ± 2.84	5.05	*P* = 0.0414	307/242
3-methyl Xanthine	3	18.53 ± 3.67/18.36 ± 3.52	0.93	*P* = 0.2218	276/238
7-methylXanthine	3	18.69 ± 3.64/17.24 ± 3.35	8.41	*P* < 0.001	220/255
1,3-dimemethyl Xan	3	18.29 ± 2.73/17.33 ± 2.99	5.54	*P* = 0.0496	268/226
1,7-dimemethyl Xan	3	18.75 ± 3.36/16.34 ± 2.66	14.75	*P* < 0.001	317/266
3,7-dimemethyl Xan	3	17.64 ± 2.46/17.79 ± 3.06	-0.84	*P* = 0.0757	204/249
Caffeine	16	20.09 ± 2.89/17.41 ± 2.91	15.39	*P* < 0.001	1855/1756

## Discussion

In this study, we showed that low concentrations of caffeine extended the lifespan of *C. elegans*. Previous studies also reported that caffeine increased the lifespan of *C. elegans*, but the doses used ranged from 400 to 4000 μg/ml (Sutphin et al., 2012), which were all higher than physiological dose. The amounts we used in this study were much lower, and we showed that as low as 10 μg/ml caffeine still possessed the longevity-promoting effect. This low concentration was more close to the physiological dose. However, worms are quite different from humans, so the dose information acquired from these results was limited.

It seemed paradoxical about whether *daf-2* was necessary for the effect of caffeine on longevity, considering the inconsistent results of *e1371* and *e1370* mutant strains. Results of previous *daf-2* RNAi and mutant (*e1370*) experiments indicated that the presence of *daf-2* was necessary for high concentration of caffeine’s effect (Bridi et al., 2015). Our results of *e1370* suggested that low concentration of caffeine’s effect was dependent on *daf-2 (e1370)*. However, low concentrations of caffeine further increased the lifespan of *daf-2* (*e1371*) mutant worms, which implied *daf-2* was not necessary. The difference was reasonable, since different *daf-2* alleles can behave very differently (Patel et al., 2008). Therefore, whether *daf-2* was crucial for the effect of low concentrations of caffeine on longevity needs further study.

Consistent with other reports, the presence of *age-1*, which acted downstream of *daf-2*, was irreplaceable in this study. There is a possibility that caffeine directly targeted *age-1* to inhibit the AKT pathway and subsequently modulated downstream gene expression. *Age-1* is a homolog of PI3K in mammals, and it has been reported that caffeine directly inhibited PI3K *in vitro* (half maximal inhibitory concentration-IC50 = 15 μg/ml for p110σ subunit) (Foukas et al., 2002). So it is possible that caffeine acted on *age-1* independent of its receptor, *daf-2*.

Caffeine is one of the most widely consumed psychoactive substances in the world (Fredholm et al., 1999). Caffeine intake is associated with increased life expectancy in human and animal models (Lublin et al., 2011; Freedman et al., 2012). Nevertheless, the disadvantages of caffeine such as sleep deprivation and addiction have restricted its use for promoting human health. We aim to change the structure of caffeine by chemical derivation to obtain novel drugs that retain the effect of caffeine on human health but which are unable to cross the blood–brain barrier. According to results in this study, the 1-methyl moiety of caffeine was irreplaceable, but 3-methyl was unnecessary. These results were of importance because we could draw the conclusion that 1-methyl was more important than 3-methyl in lifespan extension. In future studies, the derivation of caffeine should focus on replacing the 3-methyl group, as it may lead to the discovery of a novel longevity-promoting compound.

## Author Contributions

JS, XW, and CF conceived and designed the experiments. XD, YG, QH, ML, and XH performed the experiments. LY and SH conducted the *daf-2* (*e1371*) mutant worms and analyzed the data. XD and YG analyzed the data. JS, XW, and CF contributed reagents, materials, and analysis tools. XD, YG, and CF wrote the manuscript.

## Conflict of Interest Statement

The authors declare that the research was conducted in the absence of any commercial or financial relationships that could be construed as a potential conflict of interest.
