# Supplementary material for: Low Concentrations of Caffeine and Its Analogs Extend the Lifespan of Caenorhabditis elegans by Modulating IGF-1-Like Pathway
Source: Front Aging Neurosci. 2018 Jul 16;10:211. doi: 10.3389/fnagi.2018.00211 (PMC6054938; doi:10.3389/fnagi.2018.00211)
Supplement: Supplementary file 1 [file Table_1.docx]

**Supplementary Table S1 | Effects of 50 µg/ml caffeine on lifespan**

| strain | Number of experiments | Mean lifespan (days)  (+caf/-caf) | Percentage change | Number of animals  (+caf/-caf) |
| --- | --- | --- | --- | --- |
| N2 | 16 | 20.09±2.89/17.41±2.91 | 15.39 | 1855/1756 |
| daf-2(e1371) | 3 | 32.81±5.23/29.38±5.26 | 11.67 | 297/232 |
| age-1(hx546) | 4 | 26.08±3.95/25.98±4.27 | 0.38 | 336/312 |
| akt-1(ok525) | 3 | 26.84±4.06/26.78±5.13 | 0.22 | 268/226 |
| akt-2(ok393) | 3 | 27.56±3.88/27.69±4.73 | -0.47 | 216/235 |
| daf-16(mu86) | 4 | 17.24±2.05/17.26±1.97 | -0.12 | 259/333 |

**Supplementary Table S2 | Effects of 50 µg/ml caffeine and its analogues on lifespan**

| compound | Number of experiments | Mean lifespan (days)  (+caf/-caf) | Percentage change | Number of animals  (+caf/-caf) |
| --- | --- | --- | --- | --- |
| Xanthine | 3 | 17.11±2.34/16.97±2.56 | 0.82 | 230/215 |
| 1-methyl Xanthine | 3 | 16.35±2.55/17.22±2.84 | 5.05 | 307/242 |
| 3-methyl Xanthine | 3 | 18.53±3.67/18.36±3.52 | 0.93 | 276/238 |
| 7-methylXanthine | 3 | 18.69±3.64/17.24±3.35 | 8.41 | 220/255 |
| 1,3-dimemethyl Xan | 3 | 18.29±2.73/17.33±2.99 | 5.54 | 268/226 |
| 1,7-dimemethyl Xan | 3 | 18.75±3.36/16.34±2.66 | 14.75 | 317/266 |
| 3,7-dimemethyl Xan | 3 | 17.64±2.46/17.79±3.06 | -0.84 | 204/249 |
| caffeine | 16 | 20.09±2.89/17.41±2.91 | 15.39 | 1855/1756 |
